# Survivorship of total knee arthroplasty in poliomyelitis patients: long-term results from the R.I.P.O. registry and single-institution retrospective study

**DOI:** 10.1007/s00402-024-05426-y

**Published:** 2024-07-28

**Authors:** Marco Manzetti, V. Digennaro, A. Di Martino, B. Bordini, L. Benvenuti, R. Ferri, D. Cecchin, C. Faldini

**Affiliations:** 1 IRCCS – Istituto Ortopedico Rizzoli, Bologna, 40136 Italy; 2 Department of Biomedical and Neuromotor Science–DIBINEM, University of Bologna, Bologna, Italy; 3 Laboratorio di Tecnologia Medica, IRCCS Istituto Ortopedico Rizzoli, Via Giulio Cesare Pupilli, 1, Bologna, 40136 Italy

**Keywords:** Arthroplasty, Poliomyelitis, Total Knee Replacement, Survival, Genu Recurvatum, Instability, Neurological, Survivorship, Registry

## Abstract

**Introduction:**

The survival of total knee arthroplasty (TKA) in patients with poliomyelitis remains a debated topic due to the high recurrence of postoperative genu recurvatum. This study aims to report the long-term survival of TKA in patients with poliomyelitis, using data from the Italian Register of Prosthetic Implantology.

**Materials and methods:**

A registry-based population study was conducted, utilizing data from the Emilia Romagna orthopedic arthroplasty implants registry (RIPO - Registro Implantologia Protesica Ortopedica). The cohort consisted of 71 patients with poliomyelitis-related arthritis who underwent TKA. The study assessed and analyzed demographic data, implant type, fixation method, insert type, and level of constraint. Additionally, variations in preoperative and postoperative both clinical and functional Knee Society Scores (KSS) were collected.

**Results:**

Eight implants required revision surgery (16%), and three patients died (6.1%), resulting in a 10-year survival rate of 86.6% and a 15-year survival rate of 53.9%. Aseptic loosening was the primary cause of revision, accounting for 37.5% of failures, followed by insert wear (25%). No statistically significant correlation was found between the level of constraint and implant survival (p=0.0887, log-rank). Both the clinical and functional KSS improved postoperatively.

**Conclusion:**

TKA is a viable alternative to knee arthrodesis and, in properly selected patients, might represent the first-choice treatment for articular degeneration due to its high survivorship. Despite the complexity of these cases, TKA can effectively alleviate articular pain, instability, and angular deviation, thereby preserving knee functionality.

## Introduction

Poliomyelitis is an infective disease caused by Poliovirus, a serotype of the species Enterovirus C, belonging to the family of Picornaviridae [1].

There are two main clinical presentations in the case of CNS polio infection. The first one, the post-polio syndrome (PPS), is a condition that may interest patients long-time after the initial infection; it is rarely life-threatening, usually symptoms are muscle weakness, fatigue, and joint pain. The second one is acute anterior poliomyelitis (AAP), more frequent when an infection is contracted during adulthood. However, less than 1% of patients develop muscle paralisys [2], which affects more frequently the lower limbs and can occur even in a single muscle. These patients tend to develop abnormal alignment of the lower limb and muscle hypotonia, especially of the quadriceps muscle, with an associated ligamentous laxity that predisposes to knee pathologies at a young age [3]. The most common clinical features found in polio patients are: angular deformities of the metaphysis, tibial external rotation, excessive valgus, bone loss, narrow femoral and tibial canal, reduced strength of the femoral quadriceps muscle, ligamentous laxity, and finally genu recurvatum or hyperextension [3].

Clearly this biomechanical environment can lead to osteoarthritis, pain, arthrosis, ligamentous laxity and painful extension that can be corrected at first with osteotomies and soft tissue releases, but almost inevitably will require total prosthetic replacement of the affected knee [3].

Nowadays, total knee arthroplasty (TKA) has been shown to promote excellent results in terms of pain reduction, functionality, and quality of life improvement [4–8]. However, it represents a challenging procedure in these patients since they develop acquired articular and metaphyseal angular deformity, bone loss, the narrowness of the femoral and tibial canals, impaired quadriceps strength, flexion contracture, and ligamentous laxity that can influence the implant survivorship. Moreover, the abnormal osseous and non-osseous knee anatomy, i.e., abnormal lower limb rotation, predispose to a chronic dislocated patella [4]. This requires an extensive multidisciplinary planning and accessories procedures, either on bone or soft tissue, in order to give a proper implant alignment and patellar tracking.

To the best of the Authors’ knowledge, few studies have investigated the survivorship of total knee implants in patients with limbs affected by poliomyelitis [4–7] and no registry studies are available on the topic.

The present study aims to report the medium and long-term results of total knee arthroplasty (TKA) performed on patients affected by APP by analysing the follow-up data of an Italian regional registry.

## Materials and methods

This study was approved by our institution’s ethics committee (approved study PTG-P, protocol no.° 0012883) and all the subjects have given their written informed consent to publish their clinical information.

A registry-based population study has been conducted by reporting and analysing data collected by the Emilia Romagna orthopaedic arthroplasty implants registry (called RIPO for *Registro Implantologia Protesica Ortopedica*) [9–13].

The data collected include demographics of the patients, diagnosis leading to joint replacement, model and design of the implant, and the surgeon performing the procedure. At the time of surgery for a primary knee implant, the surgeon fills out a dedicated form containing information about the patient’s pathology, demographic data, date of admission, surgery, and discharge, as well as the characteristics of the prosthesis and type of fixation. This is a standard procedure for all patients undergoing prosthetic surgery in Emilia Romagna.

RIPO also collects data on any revision procedure, even if they were not performed in Emilia Romagna. In fact, every surgical procedure performed in Italy is notified and billed back to the patient’s region of residence. Thus, for revision surgery, RIPO can collect the exact same data collected for primary knee arthroplasty, plus the cause for revision surgery.

RIPO was asked to select patients affected by knee osteoarthritis secondary to APP who underwent TKA between January 1st 2000 and December 31th 2020. The sample size has not been calculated since all available cases from the RIPO registry were included in the analysis. Demographic data, implant type, type of fixation, insert type, and level of constraint were extracted and analysed together with data about implant survival. Failures were recorded up to May 2022.

Clinical data was obtained from medical records. Patients’ demographics, BMI, age at surgery, muscle function via MRC scale [14], operative time, intraoperative complication, and hospital stay were collected. A physician will usually perform a pre-operative Knee Society Score (KSS) [15] during a clinical evaluation before to a surgical procedure. During follow-up visits, postoperative KSS data were also regularly gathered.

**Fig. 1:**
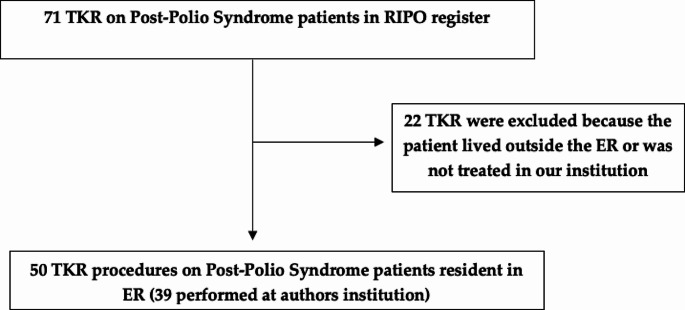
Flowchart of patient recruitment from RIPO registry and author’s institution

Since data on implant survival in patients residing outside Emilia Romagna are not collected in RIPO, implants performed on patients living outside the region and that did not undergo surgery in the Authors’ institution were excluded, to minimize bias due to loss of follow-up (Fig. 1).

Implants performed on patients living outside the region but operated in our institution were included because data could still be retrieved. Indeed, when a patient undergoes a surgical procedure has its social security number assigned to RIPO. This permits to report the registry data on primary and\or revision implant, and to add these data on respective survival curves.

Data of implanted prosthesis in polio patients were compared to the overall data of TKA’s by consulting the same registry. This permitted us to fully describe the regional trends in patients, implant types and methods of fixation, as well as survivorship.

### Statistical analysis

**Graph 1:**
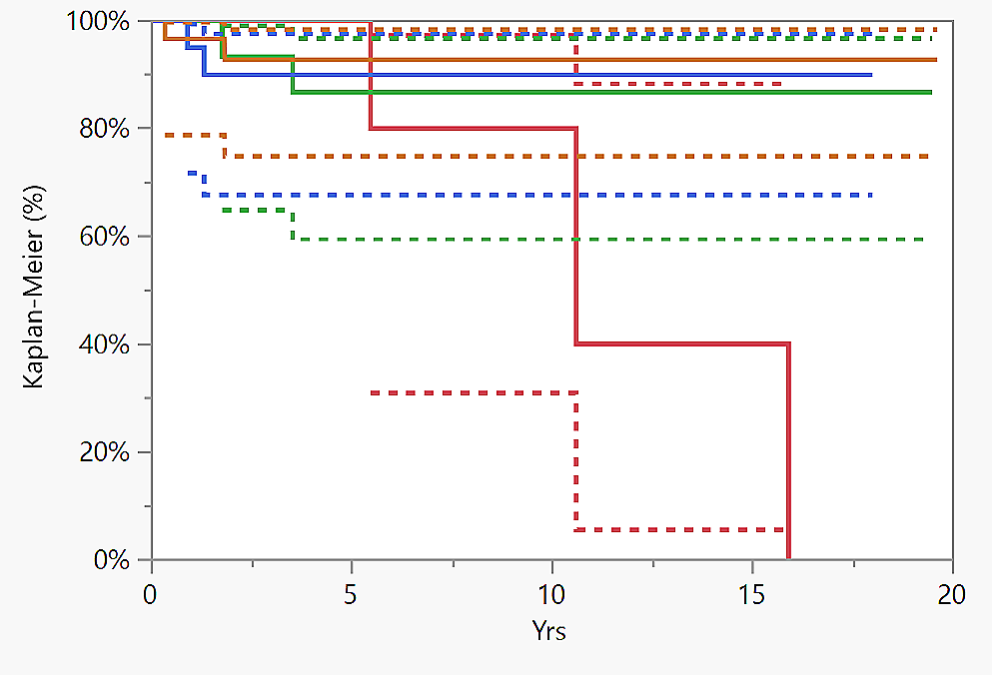
Kaplan-Meyer curve of polio patients TKA survival

**Graph 2:**
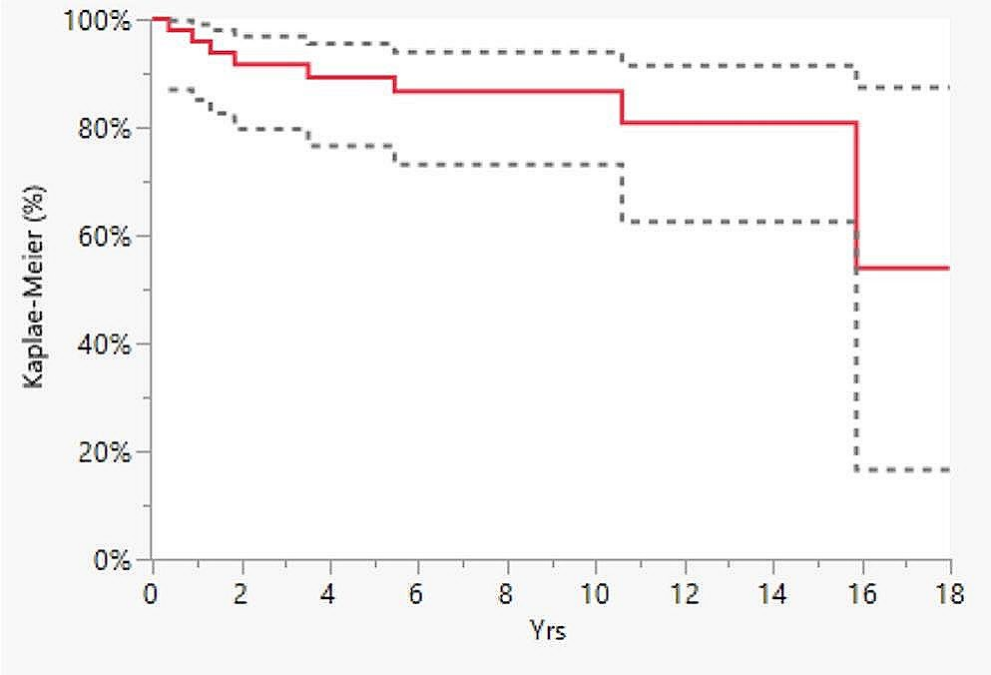
Kaplan- Meyer curve of polio patients TKA survival according to the level of constraint. The observed difference between the curves is not statistically significant (*p* = 0.0887, long-Rank). (Red: no constraint; Green: Posterior stabilized; Blue: Medial Pivot; Orange: Hinged)

Descriptive statistics were used to summarize the data, presented as median and mean with standard deviation (SD) for continuous variables and as frequency with percentage (%) for categorical variables. Statistical significance was calculated using the Mann-Whitney test for clinical quantitative data. A p-value of < 0.05 was considered statistically significant. Survival curves were calculated and plotted using the Kaplan- Meier method (Graph 1 and Graph 2). Prosthesis failure was defined as the revision of even one prosthetic component. Implants were followed until the last date of observation (date of death or date of visit).

Statistical analysis was performed using JMP, version 12.0.1. (SAS Institute Inc, Cary, NC, 1989–2007).

## Results

### Implant survival

In Emilia Romagna, 71 patients with poliomyelitis were treated from January 1, 2000, to December 31, 2020, of which 39 (55%) were treated in our institution (Table 1). These 71 prostheses represented only the 0.1% of the total TKAs implanted in Emilia Romagna (71 out of 103,807).

**Table 1:** Number of knee replacement surgeries performed in the ER region on patients resident everywhere and with admission date between 1st January 2000 and 31st December 2020

Years	*N*° of procedures	%
2000	2	2,8
2002	3	4,2
2003	3	4,2
2004	1	1,4
2005	1	1,4
2006	2	2,8
2007	3	4,2
2008	4	5,6
2009	8	11,3
2010	6	8,4
2011	4	5,6
2012	6	8,5
2013	3	4,2
2014	6	8,5
2015	6	8,5
2016	2	2,8
2017	6	8,5
2018	1	1,4
2019	2	2,8
2020	2	2,8
**Total**	**71**	**100,0**

In Emilia Romagna, a total of twenty-two on seventy-one patients (31%) were males and forty-nine on seventy-one patients (69%) were females. The average age at surgery was 62.9 for females (range 49–83) and 62.5 for males (47–81). In total, 53 out of 71 implants (73,9%) were total knee arthroplasty without patellar component, while 18 out of 71 implants were total knee arthroplasty with patellar component (26.1%).

Regarding the fixation method, most implanted prostheses were cemented (69 out of 71, 97.2%), 1 (1.4%) was uncemented and 1 out of 71 (1.4%) was cemented only on the femoral component (Table 2). The polyethylene insert was fixed in 37 cases out of 71 (52.1%) and mobile in 34 out of 71 (47.9%) (Table 3). These fixation method proportions were similar compared to the ones described for all TKAs implanted present in the registry; with 94,3% for the cemented prostheses and 3.9% for the uncemented prostheses.

**Table 2:** Distribution of failure causes in author’s institution and survival rate and confidence interval of TKAs implanted during a 15-year interval in RIPO registry

Cause of revision				Rate		% (total TKA)		% Distribution failure causes		
Total aseptic loosening				3/50		6.0		37.5		
Insert wear				2/50		4.0		25.0		
Septic loosening				1/50		2.0		12.5		
Tibial aseptic loosening				1/50		2.0		12.5		
Breakage of prosthesis				1/50		2.0		12.5		
**Total**				**8/50**		16.0		100.0		
**% survival (Confidence interval 95%)**										
**Endpoint: Any component revised**	**1Yr**	**3Yrs**	**5Yrs**		**7Yrs**		**10Yrs**		**15Yrs**	
*Polio*	*95.9* *(85.1–99.0)*	*91.6* *(79.6–96.8)*	*89.2* *(76.5–95.4)*		*86.6* *(73.0-93.9)*		*86.6* *(73.0-93.9)*		*53.9* *(16.5–87.3)*	
*Prostheses at risk*	*47*	*40*	*35*		*25*		*18*		*3*	

**Table 3:** Number of primary total knee arthroplasty operations carried out on patients with admission date between 1st January 2000 and 31st December 2020, according to fixation method, insert type and level of constraint in RIPO registry and author’s institution

Fixation (RIPO)					
	*N*.		%		
Cemented prosthesis	69		97.2		
Uncemented prosthesis	1		1.4		
Uncemented femoral component + Cemented tibial component	1		1.4		
Cemented femoral component + Uncemented tibial component	-				
Total	71		100.0		
**Insert Type (RIPO)**					
	**N.**		**%**		
Fixed	37		52.1		
Mobile	34		47.9		
Total	71		100.0		
**Constraint (RIPO)**					
	**N.total**		**%**		
No constraint	5		7,0%		
Posterior stabilized	16		22,5%		
Medial Pivot	21		29,6%		
Hinged	29		40,8%		
Total	71		100.0		
**Constraint (Institution)**					
	**Insitution N.**	**%**	**MRC**		
			**3/5**	**4/5**	**5/5**
No constraint	2	5.6%	0	0	2
Posterior stabilized	11	28%	0	6	5
Medial Pivot	11	28%	0	3	8
Hinged	15	38,4%	15	0	0
Total	39	100.0	15	9	15

The degree of constraint of the implant was variable: the most common choice was a hinged implant (29 out of 71 implants, 40.8%), followed by a medial pivot **(**21 out of 71 implants, 29.6%); a posterior stabilized (PS) implant was chosen in 16 out of 71 implants (22.5%) and a cruciate retaining (CR) in 5 out of 71 (7%) (Table 3).

A similar variability in the choice of the implant constraint was also seen in Author’s Institute, with hinged implant as the most implanted (15 out of 40, 37,5%), followed by medial pivot constraint (11 out of 40, 27,5%); a PS implant was implanted in 11 out of 40 knees (27,5%) and a CR in 2 out of 40 (5%) (Table 3).

These degree of constraint proportions were different compared to the ones described for all TKAs implanted present in the registry; with 65% for the posterior stabilized, followed by 30.1% for cruciate retaining prostheses and 4.9% for the medial pivot and hinged implants.

An assessment of muscle function was performed only in patients treated in our institution, applying MRC scale [14].

All patients had minimum 3\5 MRC score (the patient can overcome gravity and move through the full range of motion without resistance coming from the examiner) as eligible criteria to undergo surgery.

There were 15 patients with an MRC score of 3\5, all treated with a hinged implant. Nine patients showed an MRC score of 4\5, 6 were treated with a PS prosthesis and 3 with a medial pivot. Finally, 15 patients had a 5\5 MRC score, 2 were treated with no constrain implants, 5 with PS prosthesis and 8 with medial pivot implant (Table 3).

At an average follow-up of 7.6 years (range 1.4–18), 50 out of 71 prostheses implanted in Emilia Romagna completed follow-up.

Eight implants underwent revision surgery (16%) and 3 patients died (6.1%), with a 10-year survival of 86.6% and 15-year survival of 53.9% (Graph 1, Table 2). Aseptic loosening was the main cause of revision, accounting for 37.5% of failures in the cohort, followed by insert wear (25%) (Table 2). The survival rate of all TKAs implanted in Emilia Romagna was different when compared to TKAs implanted in polio patients. In fact, the 10-year survival rate was 95% and the 15-years survival rate was 94%.

Excellent stability, assessed by clinicians and self-reported by patients, was achieved in all patients, except 5 (10%) patients (2 CR, 2 PS, and 1 hinged) who reported the recurrence of genu recurvatum after an average of 1.7 years; two of them required revision surgery and one needed the use of a brace.

When adjusted for gender and age, no influence was found on the outcomes of prosthetic surgery (*p* = 0.062).

Considering the level of constraint, no statistically significant correlation was found between the level of constraint and implant survival (*p* = 0.0887, long-Rank). No cases of arthrofibrosis were reported in the RIPO (Graph 2. and Table 4).

**Table 4:** Survival rate and confidence interval of TKAs implanted during a 15-year interval according to the level of constraint

% survival (Confidence interval 95%)						
Endpoint: any component revised	1Yr	3Yrs	5Yrs	7Yrs	10Yrs	15Yrs
No constraint	*100.0*	*100.0*	*80.0* *(30.9–97.3)*	*-*	*-*	*-*
*Prostheses at risk*	*5*	*5*	*5*	*3*	*2*	*1*
Posterior Stabilized	*100.0*	*86.7* *(59.5–96.6)*	*86.7* *(59.5–96.6)*	*86.7* *(59.5–96.6)*	*86.7* *(59.5–96.6)*	*86.7* *(59.5–96.6)*
*Prostheses at risk*	*16*	*14*	*11*	*9*	*6*	*3*
Medial Pivot	*90.0* *(67.6–97.5)*	*90.0* *(67.6–97.5)*	*90.0* *(67.6–97.5)*	*90.0* *(67.6–97.5)*	*90.0* *(67.6–97.5)*	*90.0* *(67.6–97.5)*
*Prostheses at risk*	*19*	*16*	*13*	*11*	*6*	*1*
Hinged	*96.4* *(78.6–99.5)*	*92.6* *(74.7–98.1)*	*92.6* *(74.7–98.1)*	*92.6* *(74.7–98.1)*	*92.6* *(74.7–98.1)*	*92.6* *(74.7–98.1)*
*Prostheses at risk*	*27*	*24*	*22*	*15*	*12*	*2*

### Clinical and surgical outcomes

Since most of the prostheses were implanted in our institution, clinical and surgical date could be retrieved easily from clinical charts.

Forty TKAs were implanted on 39 patients, 10 males (25.6%) and 29 females (74.4%); 21 (53.8%) on the right knee, 18 (46.2%) on the left knee, and one (2.5%) bilateral case. The average follow-up was 81.6 months (range 25–242 months). At the time of surgery, the mean patient age was 65.3 years (range 41–84), and the average body mass index (BMI) was 26.6 (range 17–33). The mean operative time was 122.5 min (range 80–150 min), and the average hospital stay was 7.6 days (range 5–18 days). One patient had an intraoperative tibial shaft fracture, which required fixation with two cortical screws and no-weight-bearing for six weeks.

At the follow-up, three patients died, and one was lost. Therefore, 35 KSS questionnaires were administered at the last follow-up (average of 107.2 months, range 5–193 months). The preoperative clinical KSS improved from an average of 31.8 points (range 3–48) to a post-operative score of 74.6 points (range 60–88). The average functional KSS improved from 31.3 (range 12–60) preoperatively to 58.4 (range 30–91) postoperatively.

At the last follow-up, none of the 40 implants underwent revision.

## Discussion

The aim of the present study was to report the medium and long-term results of total knee arthroplasty performed on patients affected by APP by analysing the follow-up data of an Italian regional registry. The most important finding of the present study was that, according to our registry data, TKA in post-polio syndrome patients showed a 10-year survival of 86.6%. This percentage is in line with the results of a systematic review by Prasad [3] et al., who reported an overall survival rate of 93% at six years, and with the results reported by Rahman et al [16] (also 93% at 6 years), but results lower than the survival rate of the overall TKAs performed in Emilia-Romagna.

This can be explained considering the most common deformities and clinical manifestations found in polio patients like excessive valgus, bone loss, narrow femoral and tibial canal, reduced strength of the femoral quadriceps muscle, ligamentous laxity, and genu recurvatum. All these aspects complicates not only the implantation of the prostheses, from a technical standpoint view, but also the stability of the implant, thus reducing their survival.

The issue whether to use constrained implant is still debated, with the rationale of using hinged implants in patients with a poor quadriceps strength in order to maximize stability, survival and reduce recurrence of hyperextension and poor-long term outcomes.

In our registry we found that most of the implanted prostheses have a higher constraint, with more than 40% with hinged prostheses, with higher levels of survivorship compared to non-hinged implant. Although, this difference didn’t reach statistically significant values (*p* = 0.087) in rate of survival at long term follow-up. This finding could be influenced by relative low number of polio patients (even though this population is the largest described in literature) and with the lack of patients’ nonresident in ER.

Anyway, in our high-volume center institution, the hinge constrained implant is the best choice in polio patients in terms of rapid restoration of function and with reduced risk of recurrence of hyperextension and revision surgery.

This is consistent with the results described by Rahman et al [16], where the authors need to use a customized rotating hinge constraint with 5° of hyperextension built-in in order to avoid excessive hyperextension obtaining encouraging results. In other studies [4, 5, 7], when less constrained implants where implanted, hyperextension was avoided using a combination of techniques, such as under resection of distal femur or increased tibial slope with subsequent intraoperative soft tissue balancing.

Moreover, Tigani et al. [8]. stated that consider more constrained implants has to be encouraged in polio patients, seen that modern rotating hinge constraint can recreate a more physiological knee kinematics, reducing stress at implant-bone interface.

As for the causes of revision, aseptic loosening (AL) was the most important in our results, accounting for 37.5% of all failures, followed by insert wear (25%). The incidence of AL in our patients was 6%, which is in line with J. Gan et al [17] who reported an incidence of 5.9%. These results are not surprising, since aseptic loosening is a frequent cause of failure [18] and seems to have become the largest cause of late failure of knee arthroplasties [19, 20] surpassing infection and instability as the most common cause of late revision (> 2y) [19]. However, some other authors report instability, periprosthetic joint infection, and periprosthetic fractures to be the most frequent causes of revision of TKA in polio patients [5, 7, 8]. The reason why the causes of revision vary between studies is unclear, but it may be associated with the use of different definitions of failures among studies. In addition, twenty years ago, polyethylene wear was reported as the most prevalent failure mechanism, accounting for 56% of all revision TKAs [19], and early failures of cementless implants were reported to account for 13% [21]. These data highlight the successful development of more wear resistant biomaterials or better locking mechanism for the polyethylene into the tibial tray [22]. Instability remains a common TKA revision etiology; however the incidence has decreased possibly secondary to advancements in surgical technique and more common use of prosthetic designs utilizing a posterior stabilized construct [19].

Another important finding of the present study was the improvement of the clinical and functional KSS; the scores increased from 31.8 to 31.3 to 74.6 and 58.4, respectively.

This improvement is comparable to the data present in the literature: Rahman et al. [16]. registered an incrementation in the OKS from 10.6 to 30.7; J. Gan et al. [17]. described an improvement in average knee scores from a mean of 26.86 (0–59) preoperatively to 82.25 (62–93) postoperatively; in L. Jordan et al. [7]. paper the average Knee Society score improved from 33 points (range, 10–45 points) preoperatively to 85 points (range, 73–92 points) at last follow-up.

Therefore, the clinical and functional results along with the implant survival are encouraging. In fact, post-polio knees are extremely complex from a biomechanical point of view. For this reason, knee arthrodesis has represented the first-line treatment for knee osteoarthritis in APP patients since the mid-50s; in fact, this procedure could address both knee instability and pain, as well as severe angular deformities [23–25]. Nowadays, total knee arthroplasty (TKA) has been shown to promote excellent results in terms of pain reduction, functionality, and quality of life improvement [4–8], representing a long-lasting valuable first-line treatment that maintains knee mobility.

This study does not come without limitations. First, patient-reported outcomes and functional data were collected only in a small number of patients. Second, the registry does not collect information about the preoperative status of the operated knee. Moreover, being a retrospective study, many confounders might affect the results. Finally, the procedures were performed by different surgeons in different hospitals.

## Conclusion

In our opinion, TKA is a valid alternative to knee arthrodesis and in properly selected patients might represent the first-choice treatment for articular degeneration. This procedure in such complex cases is very challenging, but it can resolve articular pain, instability, and angular deviation preserving knee functionality; in addition, arthrodesis can still be performed in case of TKA failure.

## References

[1] Bodian D, Horstmann DM (1965) PolioViruses. In: Horsfall FL Jr TI (ed) Viral and rickettsial infections of Man, 4th edn. Lippincott Williams & Wilkins, Philadelphia, pp 430–473

[2] Mehndiratta MM, Mehndiratta P, Pande R (2014) Poliomyelitis: Historical Facts, Epidemiology, and Current Challenges in Eradication. The Neurohospitalist. 10.1177/1941874414533352 10.1177/1941874414533352 PMC4212416 25360208

[3] Prasad A, Donovan R, Ramachandran M et al (2018) Outcome of total knee arthroplasty in patients with poliomyelitis: a systematic review - PubMed. EFORT Open Rev 3:358–362 30034816 10.1302/2058-5241.3.170028 PMC6026880

[4] Patterson BM, Insall JN (1992) Surgical management of gonarthrosis in patients with Poliomyelitis. J Arthroplasty 7:419–426. 10.1016/S0883-5403(07)80034-9 1431926 10.1016/s0883-5403(07)80034-9

[5] Giori NJ, Lewallen DG (2002) Total knee arthroplasty in limbs affected by Poliomyelitis. J Bone Jt Surg - Ser A 84:1157–1161. 10.2106/00004623-200207000-00010 10.2106/00004623-200207000-00010 12107315

[6] Moran MC (1996) Functional loss after total knee arthroplasty for Poliomyelitis. Clin Orthop Relat Res 243–246. 10.1097/00003086-199602000-00033 10.1097/00003086-199602000-00033 8625587

[7] Jordan L, Kligman M, Sculco TP (2007) Total knee arthroplasty in patients with poliomyelitis. J Arthroplasty 22:543–548. 10.1016/j.arth.2006.03.013 17562411 10.1016/j.arth.2006.03.013

[8] Tigani D, Fosco M, Amendola L, Boriani L (2009) Total knee arthroplasty in patients with Poliomyelitis. Knee 16:501–506. 10.1016/j.knee.2009.04.004 19443223 10.1016/j.knee.2009.04.004

[9] RIPO (2010) Register of the Orthopaedic Prosthetic Implants. https://www.ior.it/en/curarsi-al-rizzoli/register-orthopaedic-prosthetic-implants. Accessed 1 Jul 2023

[10] Di Martino A, Bordini B, Barile F et al (2021) Unicompartmental knee arthroplasty has higher revisions than total knee arthroplasty at long term follow-up: a registry study on 6453 prostheses. Knee Surg Sport Traumatol Arthrosc 29:3323–3329. 10.1007/s00167-020-06184-1 10.1007/s00167-020-06184-1 PMC8458185 32740877

[11] Di Martino A, Coppola MAR, Bordini B et al (2021) Clinical and radiological outcomes of total hip arthroplasty in patients affected by Paget’s disease: a combined registry and single-institution retrospective observational study. J Orthop Traumatol 22. 10.1186/s10195-021-00574-y 10.1186/s10195-021-00574-y PMC7969678 33733386

[12] Castagnini F, Sudanese A, Bordini B et al (2017) Total knee replacement in Young patients: Survival and causes of Revision in a Registry Population. J Arthroplasty 32:3368–3372. 10.1016/J.ARTH.2017.05.052 28655567 10.1016/j.arth.2017.05.052

[13] Regional Register of Orthopaedic Prosthetic Implantology (2018) Overall datahip, knee and shoulder arthroplasty in Emilia-Romagna Region (Italy). Ripo 2000–2018

[14] Paternostro-Sluga T, Grim-Stieger M, Posch M et al (2008) Reliability and validity of the Medical Research Council (MRC) scale and a modified scale for testing muscle strength in patients with radial palsy. J Rehabil Med 40:665–671. 10.2340/16501977-0235 19020701 10.2340/16501977-0235

[15] Insall JN, Dorr LD, Scott RD, Scott WN (1989) Rationale of the Knee Society clinical rating system. In: Clinical Orthopaedics and Related Research 2805470

[16] Rahman J, Hanna SA, Kayani B et al (2015) Custom rotating hinge total knee arthroplasty in patients with Poliomyelitis affected limbs. Int Orthop 39:833–838. 10.1007/s00264-014-2572-y 25341952 10.1007/s00264-014-2572-y

[17] Gan ZWJ, Pang HN (2016) Outcomes of total knee arthroplasty in patients with poliomyelitis. J Arthroplasty. 10.1016/j.arth.2016.04.019 27259390 10.1016/j.arth.2016.04.019

[18] Koh IJ, Cho WS, Choi NY, Kim TK (2014) Causes, risk factors, and trends in failures after TKA in Korea over the past 5 years: a multicenter study. Clin Orthop Relat Res 472:316–326. 10.1007/S11999-013-3252-8 23982406 10.1007/s11999-013-3252-8 PMC3889422

[19] Sharkey PF, Lichstein PM, Shen C et al (2014) Why are total knee arthroplasties failing today–has anything changed after 10 years? J Arthroplasty 29:1774–1778. 10.1016/J.ARTH.2013.07.024 25007726 10.1016/j.arth.2013.07.024

[20] Lombardi AV, Berend KR, Adams JB (2014) Why knee replacements fail in 2013: patient, surgeon, or implant? Bone Joint J 96–B:101–104. 10.1302/0301-620X.96B11.34350 25381419 10.1302/0301-620X.96B11.34350

[21] Fehring TK, Odum S, Griffin WL et al (2001) Early failures in total knee arthroplasty. Clin Orthop Relat Res 392:315–318. 10.1097/00003086-200111000-00041 10.1097/00003086-200111000-00041 11716402

[22] Kurtz SM, Gawel HA, Patel JD (2011) History and systematic review of wear and osteolysis outcomes for first-generation highly crosslinked polyethylene. Clin Orthop Relat Res 469:2262–2277. 10.1007/S11999-011-1872-4/TABLES/5 21431461 10.1007/s11999-011-1872-4 PMC3126942

[23] STEWART MJ, BLAND WG (1958) Compression in arthrodesis; a comparative study of methods of fusion of the knee in ninety-three cases. J Bone Joint Surg Am 40 A:585–606. 10.2106/00004623-195840030-00008 13539085

[24] Nelson CL, Evarts CM (1971) Arthroplasty and arthrodesis of the knee Joint. Orthop Clin North Am 2:245–264. 10.1016/S0030-5898(20)31152-4 5117351

[25] Green DP, Parkes JC, Stinchfield FE (1967) Arthrodesis of the knee. A follow-up study. J Bone Joint Surg Am 49:1065–1078. 10.2106/00004623-196749060-00004 6038857

